# Nano-Composite Filler of Heteropolyacid-Imidazole Modified Mesoporous Silica for High Temperature PEMFC at Low Humidity

**DOI:** 10.3390/nano12071230

**Published:** 2022-04-06

**Authors:** Gicheon Lee, Jinsol Kim, Jungho Park, Yukwon Jeon, Jinwon Park, Yong-Gun Shul

**Affiliations:** 1 Department of Chemical and Biomolecular Engineering, Yonsei University, 50 Yonsei-ro, Seodaemun-gu, Seoul 120-749, Korea; godjackie@hanmail.net (G.L.); jinsol13@gmail.com (J.K.); vndnwind@naver.com (J.P.); 2 Department of Environmental and Energy Engineering, Yonsei University, 1 Yonseidae-gil, Wonju 26493, Korea

**Keywords:** nano-composite, heteropolyacid, imidazole, mesoporous silica, PEMFC, high temperature, low humidity

## Abstract

Nano-composite filler has received attention for the application to high temperature and low humidity polymer electrolyte membrane (PEM) in fuel cell systems. Heteropolyacids (HPAs) are one of the most attractive materials because of their conductive and thermally stable properties, but have practical limitations due to their high solubility. We investigated the stabilization of HPA on imidazole modified mesoporous silica as a nano-composite filler. The role of mesoporous silica as a support for imidazole and the distribution of chemically bonded HPA on the surface were both confirmed through physical and chemical analysis. The developed nano-composite was utilized to a PEM as a proton conducting filler, cast with commercial Aquivion^TM^ solution. Changing the HPA: imidazole ratio and HPA wt%, the composite membrane of Im10/PWA6/Si-MCM-41 (PWA 10 wt%) resulted in higher proton conductivity compared to the non-modified membrane at all operation conditions, especially at high temperature (140 °C) and low relative humidity (RH 10%), with values of 0.3530 and 0.0241 S/m, respectively. A single cell test at H_2_/Air also showed the effect of adding the nano-composite filler at a wide range of temperatures, which outperformed a single cell with a pristine membrane even at an extremely low humidity condition.

## 1. Introduction

Fuel cells have been considered as candidates for new energy systems that can solve energy and environmental problems. A polymer electrolyte membrane fuel cell (PEMFC) is one that can directly convert chemical energy into electrical energy and has an advantage of high energy density, thus is considered as a means to replace the fossil fuel engine. In addition, PEMFC is run by a clean energy source, hydrogen, and releases water as the only by-product [1]. However, in order to apply PEMFC to small vehicles or mobile devices, there are hurdles that require simplifying the system and reducing the volume [2]. Recently, research has been conducted on the goal of simplifying water management systems and lowering material costs by operating fuel cells at high temperature and low humidity [2,3]. While the existing system requires two different heaters for each cell and steam reformer, a single heater is enough for a fuel cell system that increases the operating temperature of PEMFC. Moreover, a hydration system is not required because the humidity of the inlet gas is low. Therefore, improving the performance of PEMFC at high temperature and relatively low humidity is a new challenge.

The most important factor in PEMFC performance depends on the membrane [4]. Perfluorosulfonic acid (PFSA)-based organic polymer membranes (e.g., Nafion™), the most commonly used in PEMFC, have an excellent performance and stability because of their reliable proton conductivity and mechanical stability, especially under medium-temperature (up to 80 °C) and humid conditions. However, PFSA membranes often perform unsatisfactorily, particularly as a result of weakened mechanical stability and membrane dehydration, which causes a significant loss in proton conductivity leading to substantial reduction of power generated by the PEMFC system [5]. Therefore, in order to accelerate the commercialization of PEMFC, it is necessary to develop a new proton conductor that is not sensitive to temperature and humidity.

Heteropolyacid (HPA) is one of the most attractive inorganic materials because crystalline materials are highly conductive and thermally stable. Phosphotungstic acid (PWA), silicon tungstic acid, and phosphomolybdic acid have already been studied as additives for fuel cell membranes and have been recognized for their high potential [6,7,8,9,10,11,12,13,14,15,16,17,18,19,20,21,22]. However, heteropolyacid itself is limited in use as a material for PEMFC due to its high water solubility. Hence, the development of a water insoluble heteropolyacid with excellent proton conduction is receiving a lot of attention in the research fields. There have been some studies to control the solubility and acidity of water by replacing hydrogen in HPA with cesium, zirconium, ammonium, and other inorganic substances [22]. Among the various candidate groups, heterocyclics are also known to be capable of modifying heteropolyacid, which is not soluble in water. In particular, imidazole is one of the heterocycle containing two nitrogen atoms capable of delivering protons, and nitrogen is protonated in the imidazole system, and acts as a proton donor to the next closest imidazole when the region contains excess protons [23,24,25]. The insolubility of the imidazole and heteropolyacid composite has already been demonstrated through our previous studies and was proved by the chemical bonding between them [26,27].

In this study, in order to manufacture a PEMFC membrane for the use in high temperature and low humidity environments, a proton conduction nano-additive immobilized with heteropolyacid was utilized. For the purpose of high distribution of heteropolyacid bonded with imidazole, a support obtained by fixing imidazole to Si-MCM-41 was synthesized by a chemical anchorage technique. The nano-composite membrane was prepared by mixing the synthesized particles with commercial ionomer. Instead of Nafion™, Aquivion™ is used at high temperatures due to its better performance with lower ohmic loss resistance, hydrogen cross-over, better electro catalytic activity and stability due to its higher crystallinity, and stronger polymer structure [28,29]. In order to evaluate the membrane performances, AC impedance tests to measure proton conductivity and single PEMFC tests were performed at different temperatures (75–140 °C) and low humidity (RH 10%)

## 2. Materials and Methods

### 2.1. Materials

The chemicals used for the synthesis are as follows. Sodium Hydroxide (Lab grade, Duksan, Korea), Ludox HS-40 (Sigma Aldrich, St. Louis, MO, USA), Cetyl-trimethyl-ammonium bromide (Sigma Aldrich, St. Louis, MO, USA), Acetic acid (≥99%, Duksan, Korea), Ethanol (99.9%, Duksan, Korea), Hydrochloric acid (extra pure grade, Duksan, Korea), Dichloromethane (≥99.5%, ACS grade, Sigma Aldrich, St. Louis, MO, USA), 3-chloropropyl trimethylsilane (≥97%, Sigma Aldrich, St. Louis, MO, USA), imidazole (99%, Alfa Aesar, USA), Toluene (extra pure grade, Duksan, Korea), n-propyl alcohol (extra pure grade, Duksan, Korea), and phosphotungstic acid (Kanto Chemical, Tokyo, Japan).

### 2.2. Membranes Preparation

#### 2.2.1. Preparation of Mesoporous Silica (Si-MCM-41)

Si-MCM-41 powders were prepared according to the hydrothermal procedure proposed by Ryoo, R. et al. [30]. An amount of 3.645 g of cetyltrimethylammonium bromide (CTAB) was dissolved in 42 mL of DI water. While the solution was stirred, it was heated until the solution became transparent, then cooled. After cooling, 15 g of sodium silicate solution (Na:Si = 0.5, 20 wt%) was added dropwise, and then the mixture was placed in an oven for one night. The molar ratio of the individual components of the reaction mixture was CTAB:H_2_O:Na-Silicate = 1:300:4.96. After completing the dissolution, 50 wt% of acetic acid was added until the pH value of the solution reached 10, and it was then left in the oven for 48 h. After filtrating and washing the solution with DI water, the precipitate was dried in the oven at 100 °C. The precipitate was then filtered and washed again with ethanol/HCl solution and dried at 100 °C. At the end, the product was calcined at 600 °C for 5 h.

#### 2.2.2. Chemical Anchorage of Imidazole

At first, functionalization of Si-MCM-41 was performed for the chemical anchorage of imidazole. After adding 1 g of Si-MCM-41 to 20 mL of dichloromethane in an anhydrous argon atmosphere, 3-chloropropyl trimethoxysilane(3CP-TMS) was added as a functionalizer. The suspension was stirred for 24 h, filtered using dichloromethane and ethanol, and washed. The precipitate was dried overnight in an oven. Then, imidazole anchorage to the functionalized Si-MCM-41 was performed in toluene reflux according to the procedure of Armatas et al. [31]. One gram of functionalized powder was added to 20 mL of toluene, and a certain amount of imidazole was added to the suspension. After boiling the mixture in reflux for 24 h, the powder was filtered, washed with toluene and ethanol, and dried at 80 °C overnight.

#### 2.2.3. Immobilization of Heteropolyacid

The immobilization of heteropolyacid was carried out in the process of aqueous functionalization. First, phosphotungstic acid (PWA) was refluxed in an argon atmosphere for 24 h in a mixture of 1 g of Im(x)/Si-MCM-41 and 20 mL of toluene. The produced powder was filtered, washed with toluene and ethanol, and dried overnight. The sample was named Im(x)/PWA(y)/Si-MCM-41. x represents the amount of functionalized imidazole in mmol units, while y represents the amount of immobilized PWA by mmol per 1 g of Si-MCM-41. The weight percentage of the immobilized PWA was varied, at 10, 20, and 30%, denoted as (xx wt%).

#### 2.2.4. Composite Membrane Preparation

To manufacture the composite membranes, 24 wt% of a commercial Aquivion™ (USA) ionomer solution, was firstly added to n-propyl alcohol solvent. Then, Im(x)/PWA(y)/Si-MCM-41 powder was added to the solution and stirred strongly until the powder spread to the solution. The amount of Im(x)/PWA(y)/Si-MCM-41 was determined according to the net weight percentage of PWA. The resulting viscous solution was cast on a clean glass plate and dried in an oven at 60 °C to obtain the desired film with a thickness of 50 µm. Non-modified Aquivion™ membrane with the same procedure and thickness was prepared for comparison.

### 2.3. Characterization

Nitrogen adsorption at −196 °C was carried out with Belsorp Mini-II (Osaka, Japan) for determining surface areas, pore volumes, and pore diameters of the powders. Prior to the measurement, the sample was kept in a vacuum at 100 °C for 3 h. The Brunauer–Emmet–Teller (BET) method was used to calculate a specific surface area. The pore diameter was derived from the isothermal adsorption branch using the Barrett–Joyner–Halande (BJH) method. The pore volume was estimated from the amount of gas adsorbed at relative pressure, p/p_0_ = 0.99. X-ray diffraction (XRD) was measured by the Multiplex (Rigaku, Japan) at room temperature in the range of 2θ = 2–20° using CuKα radiation (λ = 1.5406 Å). The Fourier-transform infrared spectroscopy (FT-IR) spectrum of the powder was recorded in attenuated total reflection mode on diamond crystals using the Vertex70 (Bruker, Germany) in the range of 400–4000 cm^−1^. Field emission scanning electron microscope (FE-SEM, JSM-6701F, JEOL, Tokyo, Japan) was used to investigate particle size and shape of the powder.

### 2.4. Conductivity and Single Cell Performance

The proton conductivity of the developed membranes was measured using DC current measurement (source meter series 2400, Keithley, Cleveland, OH, USA) by a 4-probe method. Hydration steps were not performed to measure proton conductivity under severe conditions of high temperature and low humidity. The non-hydrated membrane was organized into 35 × 5 mm^2^ and then mounted on a conductivity cell (BT-112, BekkTech, Southern Pines, NC, USA) equipped with Pt foil contact. An AC Impedance spectroscopy analyzer (PGSTAT-20, Autolab, EcoChemie, Utrecht, Netherlands) was used to observe the resistance of the membranes in the frequency range of 0.1–10,000 Hz at an amplitude of 10 mV. Impedance was measured at a constant humidity of 10% RH in the temperature range of 75–140 °C, after stabilizing the membranes for 2 h at each operating condition. Experiments were repeated 3 times to confirm reproducibility, and reliability was confirmed by performing a variation of less than 5%.

In a single PEMFC test by H_2_/Air, a selected membrane with Im10/PWA6/Si-MCM-41 (10 wt%) was used. Membrane electrode assembly (MEA) was manufactured by a catalyst-coated membrane method with 0.4 mg cm^−2^ of Pt/C catalyst (40 wt%) both to the cathode and anode on the active area of 1 cm^2^. Polarization curves and impedance spectra at 0.6 V were obtained using fuel cell test station (BekkTech, Southern Pines, NC, USA) at the temperature range of 75–140 °C with a constant humidity of 10% RH, as the same conditions as proton conductivity measurements. H_2_, served as a fuel, and Air passed through the humidifier to the anode and cathode by a flow rate input of 100 mL min^−1^ and 150 mL min^−1^ with a stoichiometry of 1/1.5 (H_2_/O_2_), respectively, without giving a back-pressure, then entered the unit cell inlets. For hydration and to stimulate the catalytic activity, an activation step with constant voltage at 0.6 V for 3 h is needed. For comparison, an MEA prepared by a pristine Aquivion™ membrane was performed under the same conditions.

## 3. Results and Discussion

### 3.1. Schematic Procedure

**Figure 1:**
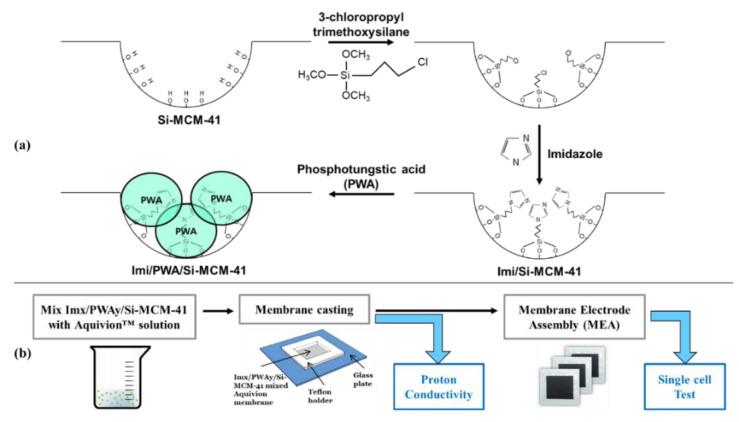
Summary of the order of (**a**) synthesizing Im(x)/PWA(y)/Si-MCM-41. (**b**) Membrane manufacturing and performance test.

Figure 1 shows the schematic diagram of the preparation method of the developed Im(x)/PWA(y)/Si-MCM-41 nano-composite filler and the modified membranes to investigate the electrochemical properties. The synthesis process basically starts with the as-synthesized Si-MCM-41 that has the role of a support to fix PWA on the surface. First in Figure 1a, 3CP-TMS is chemically attached on the Si-MCM-41 surface in an anhydrous condition, as for the functional sites. Subsequently, after replacing the functional chlorides with imidazole molecules in a toluene environment, PWA is stabilized and distributed on the Im(x)/Si-MCM-41 surface by the chemical interaction with the non-bonding N in the imidazole molecule. The final configuration of the Im(x)/PWA(y)/Si-MCM-41 nano-composite filler is presented in Figure 1a with strongly bonded PWA on the Si-MCM-41, which provides controllable insolubility. 

To investigate the effect of our filler, as shown in Figure 1b, the prepared powder is uniformly mixed with the Aquivion™ ionomer solution, cast evenly on a glass plate, and dried to produce a fine polymer electrolyte membrane for electrochemical evaluations, such as conductivity and PEMFC single cell tests under various temperatures and low humidity.

### 3.2. Characterization of Im(x)/PWA(y)/Si-MCM-41 Nano-Composite

#### 3.2.1. Nitrogen Adsorption Studies

**Figure 2:**
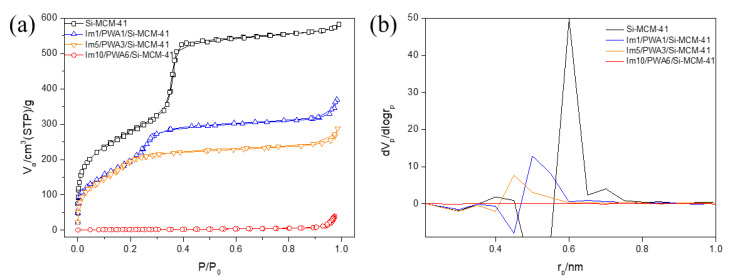
(**a**) Nitrogen adsorption–desorption isotherm. (**b**) MP Plot of Im(x)/PWA(y) modified Si-MCM-41.

The nitrogen sorption isotherms for mesoporous silica, and each composition of Im(x)/PWA(y)/Si-MCM-41 were carried out by the BET method at 77 K as illustrated in Figure 2. Table 1 summarized the calculated numeric data of the surface area, pore volume, and average pore diameter of the powder through BJH and MP methods. Figure 2a shows the nitrogen adsorption–desorption isotherms of the prepared samples. It shows that the area that N_2_ can adsorb decreases, as the content of imidazole and PWA increases.

From Table 1, the surface area of mesoporous silica (1014 m^2^/g) decreased from 880 m^2^/g in the presence of Im1/PWA1 to 6 m^2^/g in the presence of Im10/PWA6 with lower pore volumes by increasing the amount of organic and inorganic materials. In particular, the experimental results of Im10/PWA6/Si-MCM-41 show that there are only a few pores on the surface of MCM-41. Figure 2b shows the pore size distributions of the imidazole modified PWA samples onto Si-MCM-41. As the ratio of imidazole and PWA increased, the pore size dramatically decreased, and it seems that pores no longer exist at Im10/PWA6/Si-MCM-41. The changes in the surface properties of mesoporous silica may be due to the covering of a fixed imidazole group and PWA on the surface, indicating that the large pore volume at the Si-MCM-41 may provide a significant role of distributing PWA.

#### 3.2.2. FE-SEM Images

**Figure 3:**
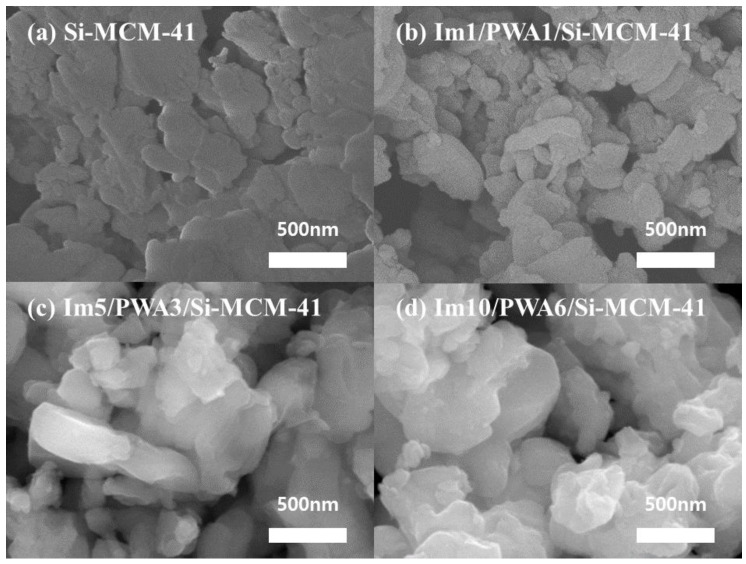
FE-SEM images for (**a**) Si-MCM-41, (**b**) Im1/PWA1/Si-MCM-41, (**c**) Im5/PWA3/Si-MCM-41, and (**d**) Im10/PWA6/Si-MCM-41.

Figure 3 shows the SEM results to investigate the morphology of the prepared Im(x)/PWA(y)/Si-MCM-41 nano-composites compared to the pristine Si-MCM-41. It can be seen that Im(x)/PWA(y)/Si-MCM-41 types of particles show a crystalline shape in a polyhedron structure with a uniform size distribution of approximately 200–500 nm. This indicates that the addition of heteropolyacid and heterocyclic materials to the Si-MCM-41 support can shape composite nanoparticles in a certain morphology through a new chemical bonding. As all of the prepared composites are uniformly dispersed as a whole, it may be seen that particles are slightly agglomerated as the imidazole-PWA content increases. It can be inferred that the size of the grain particle increases due to the increase in layer-by-layer organic and inorganic composite substances, but keep the crystalline shape in a polyhedron structure. 

#### 3.2.3. XRD Patterns

**Figure 4:**
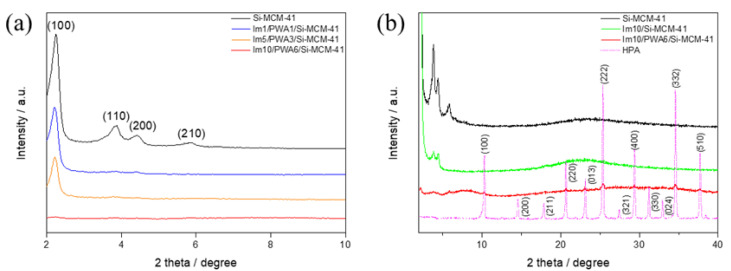
(**a**) XRD patterns of Im(x)/PWA(y) modified Si-MCM-41 and (**b**) XRD patterns of Im10/PWA6/Si-MCM-41.

Figure 4a shows the XRD patterns for the pristine Si-MCM-41, Im(x)/PWA(y)/Si-MCM-41 with specific imidazole and PWA loadings. The pure Si-MCM-41 exhibits crystalline phase characteristics such as (100), (110), (200), and (210) at the 2~7° range of 2 theta. As the amount of loaded PWA increased, the characteristic peaks of Si-MCM-41 in the Im(x)/PWA(y)/Si-MCM-41 samples decreased and even disappeared at Im10/PWA6/Si-MCM-41 due to the result of covering mesoporous silica with organic–inorganic materials. Figure 4b shows details of the XRD patterns for the pristine Si-MCM-41, Im10/Si-MCM-41 and Im10/PWA6/Si-MCM-41 in order in which the experiment was conducted. Comparing with the pristine PWA, Im10/Si-MCM-41 shows a large decrease in the main peaks of Si-MCM-41 and further disappeared after fixation of PWA. The Si-MCM-41 peaks were replaced by PWA XRD peaks assigned to the cubic Keggin structure of PWA, which we can estimate that the surface of Im10/PWA6/Si-MCM-41 is covered with organic–inorganic materials.

#### 3.2.4. FT-IR Spectra

**Figure 5:**
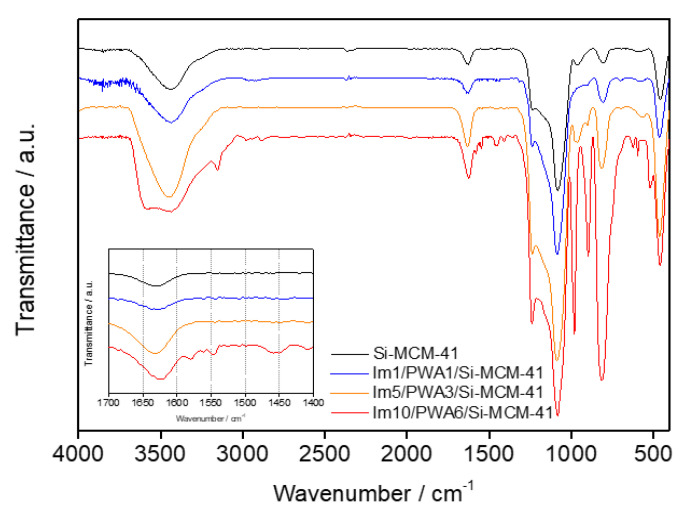
FT-IR spectra for Im(x)/PWA(y)/Si-MCM-41.

Figure 5 shows the FT-IR spectra for pristine Si-MCM-41, PWA, and different ratio of Im(x)/PWA(y)/Si-MCM-41.

Some of the important characteristic IR bands observed in the spectra are summarized in Table 2. In Im(x)/PWA(y)/Si-MCM-41, the basic IR bands of pristine Si-MCM-41 at 808–813 cm^−1^ (symmetric stretching frequency of Si-O-Si), 960–965 cm^−1^ (stretching frequency of Si-O-H), and 1000–1200 cm^−1^ (antisymmetric stretching of Si-O-Si) are observed. At the same time, Im(x)/PWA(y)/Si-MCM-41 shows characteristic peaks of PWA at 1082–1085 cm^−1^ (stretching frequency of P-O in the central PO_4_ tetrahedron), 979–981 cm^−1^ (terminal bands for W=O in the exterior WO_6_ octahedron), 896–902 cm^−1^ and 808–813 cm^−1^ (W-O-W bands) [32]. The bands observed in the range of 2800–2950 cm^−1^ are due to the propyl group present in Im(x)/PWA(y)/Si-MCM-41. A peak at 1448 cm^−1^ is detected which is assigned to the C=N of the imidazole group, and a small band between 1400 and 1410 cm^−1^ is the characteristic band of the C-N bond which can be attributed to anchoring of imidazole to the propyl group [33]. These results indicate spectra for a fine nano-composite where PWA, imidazole, and Si-MCM-41 are chemically bonded to each other. Additionally, peaks in the region of 3500 cm^−1^ are revealed corresponded to adsorbed water present in the system, which could influence to the proton conductivity at a low humidity.

### 3.3. Conductivity and Single Cell Performances of PEMFC Membrane Containing Im(x)/PWA(y)/Si-MCM-41 Nano-Composite Filler

#### 3.3.1. Proton Conductivity

**Figure 6:**
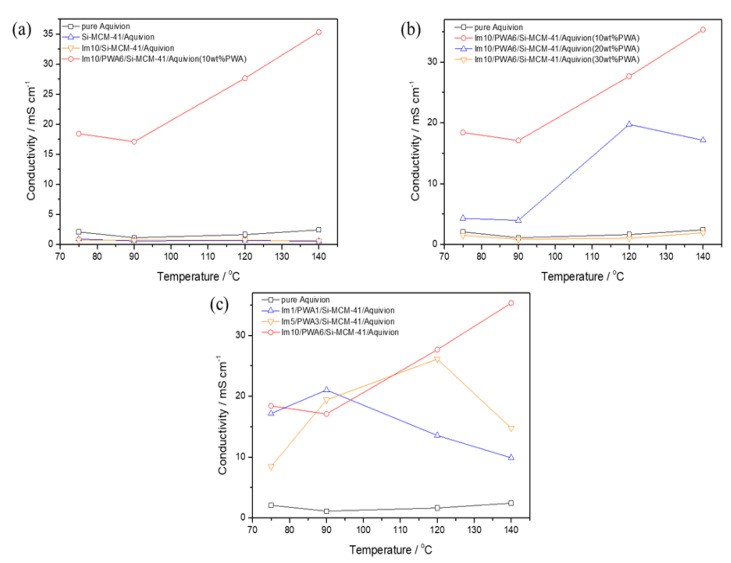
Proton conductivity at different temperatures and 10% RH of (**a**) Aquivion™, Si-MCM-41, Im10/Si-MCM-41, Im10/PWA6/Si-MCM-41 composite membranes, (**b**) Im10/PWA6/Si-MCM-41 composite membranes changing the content of Imi/PWA/MCM-41, and (**c**) 10 wt% Im(x)/PWA(y)/Si-MCM-41 composite membranes.

All experiments are operated in a temperature range of 70~140 °C and low humidity operation of 10% RH to measure the conductivity and stability of the composite membranes in harsh conditions. The conductivity results are summarized in Table 3 and plotted in Figure 6 to explain the effects of each component of Im(x)/PWA(y)/Si-MCM-41 nano-composite filler. 

As shown from the results, the membranes with Si-MCM-41 and Im10/Si-MCM-41 in Aquivion™ showed relatively lower conductivity of about 0.5 mS/cm than pure Aquivion™ of about 2 mS/cm at all temperature ranges. This means that imidazole and mesoporous silica provide no proton conducting contribution, as with an insulator. In particular, imidazole in the membrane showed a very small effect on the conductivity. Although nitrogen in imidazole can be a proton donor to the next closest imidazole when the region contains excess protons, functionalized imidazole can have limitations as reported by Marschall, R. et al. [24]: One general problem using imidazole as protogenic group is that imidazole itself, in contrast to acid groups, e.g., SO_3_H group, offers only a small amount of mobile charge carriers via self-dissociation. Furthermore, in immobilized imidazole systems, excess protons must be provided externally or by introducing much more water. The second reason is the imidazole anchorage via the nitrogen atom. For a proton transport, the one remaining free nitrogen in the imidazole ring has to pick a proton and then the group has to rotate and collide with a free nitrogen atom of another imidazole group. This process needs higher activation energy than proton transport via the ring system due to the well-known imidazole resonance. Therefore, imidazole anchored in Si-MCM-41 showed the same proton conductivity as when only Si-MCM-41 was used because there was no free nitrogen atom capable of transporting protons. This means that imidazole will only play a bridging role between PWA and Si-MCM-41. On the other hand, PWA can be the conducting role due to its high conductivity to support Aquivion™ at high temperatures and low humidity. The conductivity of a composite membrane with added PWA is significantly higher than that of other composite membranes without PWA, and nearly 17 times higher conductivity results than pure equivalent at 120 °C (Table 3).

The results of confirming the relationship between the content of Imi/PWA/MCM-41 nano-composite filler and conductivity in the membrane are shown in Figure 6b. The composition of the composite used was Im10/PWA6/Si-MCM-41, which showed the highest conductivity. In this experiment, it can be confirmed that the conductivity decreases as the content of the composite increases. Even when the PWA, a high conductive material, content of the nano-composite filler reached to 30 wt%, the conductivity was almost the same as that of when only Aquivion™ was used. Tominaga, Y. et al. [34] had reported that the inorganic content in Nafion™ can reduce the conductivity due to the increase in the insulative phase of mesoporous silica domains in the conductive Nafion matrix. When the content of PWA reaches to 30 wt%, the conductivity of the membrane is even worse than that of pure Aquivion™ membrane. The same trend is shown in the Im5/PWA3/Si-MCM-41 composite membrane listed in Table 3. This decline may be caused by an increase in the insulative phase, which consists of the agglomerates of inorganic material, where ionic transport is prevented in the conductive Aquivion™ matrix. PWA is generally disposed of in the wall and varies in volume and surface area depending on the content of Si-MCM-41. Although the content of PWA is high, it is determined that an excessive amount of insulating material of Si-MCM-41 occupying most of the volume has a negative effect on the conductivity. As a result, the content of Imi/PWA/MCM-41 nano-composite filler is optimized by 10 wt% in the prepared PEMFC membrane. 

As shown in Figure 6c, comparisons were conducted to figure out the relationship between the amount of Imidazole/PWA anchored to MCM-41 and proton conductivity. Samples with different contents of a total of three types were made with the ratio of 1:1, 5:3 and 10:6 for imidazole and PWA. As the results, three types of samples did not have a significant difference in conductivity up to 75 °C and 90 °C, but it can be seen that the conductivity of Im1/PWA1/Si-MCM-41 composite membrane dropped sharply when it reached to 120 °C. Subsequently, the conductivity of Im5/PWA3/Si-MCM-41 composite membrane tended to decrease, when it was raised to 140 °C. Among all, the conductivity of Im10/PWA6/Si-MCM-41 composite membrane, which has the highest content of PWA and imidazole, showed the highest value and even no decrease at high temperature. Aparicio, M. et al. [35] already reported that PEMFC membrane containing silica showed a decay in conductivity due to dehydration of the silica in the membrane at the temperature of 120–130 °C, which suggested the reason of the performance decline. When the conductivity started to decline, the two samples with low PWA content could not prevent dehydration from the elevated temperature. In agreement with the nitrogen adsorption studies, Im10/PWA6/Si-MCM-41 with the highest PWA content consequently prevented clogging of MCM-41 pores due to overlapping of PWA. This can be conveyed to the connection between Imidazole/PWA and silica, which may prevent the moisture from evaporating in the silica pore. Therefore, the optimization of the imidazole modified PWA can play a role in preventing the dehydration effect of the silica surface, resulting in an increase in conductivity at high temperature and very low humidity.

#### 3.3.2. Evaluation of Single Cell Performance

**Figure 7:**
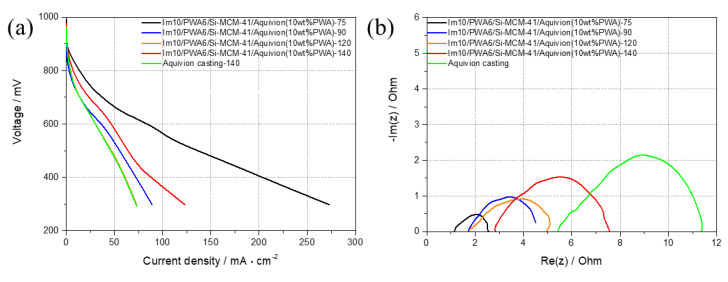
Single PEMFC performance of the Im10/PWA6/Si-MCM-41 composite membrane at 75–140 °C, 10% RH condition at H_2_/Air, plotted by (**a**) polarization curves and (**b**) impedance spectra with a reference of Aquivion^TM^ casting membrane tested at 140 °C and same conditions.

Through the highest proton conductivity, Im10/PWA6/Si-MCM-41 was selected as a nano-composite filler and casted with Aquivion™ to produce a PEMFC composite membrane for a single cell test. Figure 7a illustrated below is the results of a polarization curve in which the Im10/PWA6/Si-MCM-41 composite membrane is used at a constant temperature between 75 °C and 140 °C under 10% relative humidity conditions. As from the results at all temperatures, the composite membrane exhibited gradually decreases in performance as the temperature rises to 120 °C and then tends to increase as the temperature rises, which is similar to that of proton conductivity due to the effect of the conducting nano-composite filler. As our target of operating at high temperature and low humidity, it shows the possibility of being able to operate at high temperatures through a tendency to recover at 140 °C. The performance with the value of 45 mA/cm^2^ at 600 mV was higher than the single Aquivion™ membrane with the value of 31 mA/cm^2^ at 600 mV. The proton-conducting performance of single cell was also studied by electrochemical impedance spectroscopy (EIS).

Figure 7b shows the Nyquist plots of Im10/PWA6/Si-MCM-41 composite membrane and pure Aquivion™ membrane as a reference at selected temperatures and 10% relative humidity. The membrane resistance of the Im10/PWA6/Si-MCM-41 composite membrane slightly increased by increasing the temperature, which is probably due to a small decrease in H_2_O molecules in the composite membrane itself at very low humidity. However, the single cell performance generally included many other factors. Focusing on the working condition of 140 °C, the impedance spectra of the composite membrane show much lower membrane resistivity than the pure Aquivion™ casted membrane. Since the Aquivion™ template membrane cannot maintain H_2_O molecules inside the membrane, the resistance is much higher than that of the composite membrane containing water molecules. Therefore, it is evidence of lowering the proton transport barrier of the membrane using the modified PWA material. As a result, the composite membrane containing PWA under the same conditions has more water molecules, so it can show reasonable performance in a high temperature and low humidity environment.

## 4. Conclusions

In this study, we prepared an Aquivion™ composite membrane with a nano-composite filler of chemically anchored PWA with imidazole on Si-MCM-41, available to PEMFC applications under harsh conditions of high temperature and low humidity. An Im(x)/PWA(y)/Si-MCM-41 nano-composite filler was synthesized by chemically fixing imidazole to Si-MCM-41 and PWA onto that imidazole with an ion exchange method. As the PWA load increased, the pore size of Si-MCM-41 decreased, and pores at Im10/PWA6/Si-MCM-41 were not observed. In the XRD pattern, only the Si-MCM-41 structure appeared when the PWA content was 3 mmol, and the PWA structure pattern appeared when the PWA content reached 6 mmol. All characteristics of Im10/PWA6/Si-MCM-41 show a result of blocking the pores of Si-MCM-41 as the content of PWA increases. In the FT-IR spectrum, all characteristic peaks of PWA, imidazole, and Si-MCM-41 were observed, confirming the unique chemically bonded nano-composite structure of Im(x)/PWA(y)/Si-MCM-41. 

A composite membrane was prepared by mixing the synthesized material with a commercial Aquivion™ ionomer solution to study the effectiveness of the prepared nano-composite filler through electrochemical measurements. The content of PWA contributed to the improvement of proton conductivity of commercial Aquivion™ under harsh conditions. In particular, the composite membrane composed of Im10/PWA6/Si-MCM-41(10 wt%) showed the highest performance, which is presumed due to the ability of PWA to possess at least six water molecules even at low humidity that acts as proton conductor by the Grottuss mechanism. The polarization curve for the single PEMFC test showed an improvement in the performance of the composite membrane under severe conditions of 140 °C and RH 10%. The impedance spectrum of the composite membrane also had a lower resistivity than that of the Aquivion™ casted membrane. Heteropolyacid compounds in membranes can have large amounts of moisture and thus move protons smoothly for high conductivity, but their use has been limited due to their inherent characteristics, such as water solubility. Thus, it is possible to suggest the possibility of stabilizing heteropolyacid as a nano-composite filler in a membrane in a PEMFC under high temperature and low humidity conditions.

## Figures and Tables

**Table 1:** Surface analysis of Im(x)/PWA(y)/Si-MCM-41.

Entry	Total Pore Volume (cm^3^/g)	S_BET_ (m^2^/g)	Average Pore Diameter (nm)
Pure Si-MCM-41	0.8943	1014.5	3.4565
Im1/PWA1/Si-MCM-41	0.5939	880.89	2.7383
Im5/PWA3/Si-MCM-41	0.4453	705.02	2.5266
Im10/PWA6/Si-MCM-41	0.0610	6.7194	-

**Table 2:** FT-IR spectra Peak Assign for Im(x)/PWA(y)/Si-MCM-41.

Vibration Mode Assignment	Si-MCM-41	Im1/PWA1 /Si-MCM-41	Im5/PWA3 /Si-MCM-41	Im10/PWA6 /Si-MCM-41
Si-O	459.55	459.55	459.55	459.55
Si-O-Si /W-O-W	806.21	808.14	815.85	813.93
W-O-W	-	900.72	902.65	896.86
Si-O-H	965.80	-	962.44	-
W-O	-	979.39	975.94	981.72
Si-O/P-O	1083.95	1085.88	1087.81	1082.59
C=N	-	1448.48	1448.49	1450.41
O-H deformation	~1600	~1600	~1600	~1600
Propyl group	-	2800–2950	2800–2950	2800–2950
Absorbed H_2_O	3400–3600	3400–3600	3400–3600	3400–3600

**Table 3:** Proton conductivity of Im(x)/PWA(y)/Si-MCM-41 and Aquivion™ composite membranes (mS/cm).

Composite Membrane	75 °C	90 °C	120 °C	140 °C
Pure Aquivion™	2.0762	1.1149	1.6356	2.4104
Si-MCM-41 (inorganic 13 wt%)	0.9104	0.5651	0.6646	0.5381
Im10/Si-MCM-41 (inorganic 13 wt%)	0.7442	0.6879	0.7144	0.6061
Im10/PWA6/Si-MCM-41 (PWA 10 wt%)	18.4112	17.0879	27.6482	35.3058
Im10/PWA6/Si-MCM-41 (PWA 20 wt%)	4.2990	3.9622	19.7418	17.1270
Im10/PWA6/Si-MCM-41 (PWA 30 wt%)	1.4999	0.8503	1.0281	1.9279
Im5/PWA3/Si-MCM-41 (PWA 10 wt%)	8.4492	19.3881	26.1224	14.7610
Im5/PWA3/Si-MCM-41 (PWA 20 wt%)	9.9214	24.2919	6.0827	2.2217
Im5/PWA3/Si-MCM-41 (PWA 30 wt%)	17.0014	17.6114	3.2861	1.0849
Im1/PWA1/Si-MCM-41 (PWA 10 wt%)	17.1490	21.0177	13.5502	9.8843

## Data Availability

The data presented in this study are available on request from the corresponding author.
